# Advancing frontiers in rheumatic and musculoskeletal imaging

**DOI:** 10.1186/s12891-021-04101-2

**Published:** 2021-02-28

**Authors:** Domenico Albano, Francesco Carubbi

**Affiliations:** 1 IRCCS Istituto Ortopedico Galeazzi Milano, Via Riccardo Galeazzi 4, 20161 Milan, Italy; 2 Sezione di Scienze Radiologiche, Dipartimento di Biomedicina, Neuroscienze e Diagnostica Avanzata, Università degli Studi di Palermo, Via del Vespro 127, 90127 Palermo, Italy; 3 Department of Life, Internal Medicine and Nephrology Unit, Health & Environmental Sciences, University of L’Aquila, L’Aquila, Italy; 4 Department of Medicine, ASL 1 Avezzano-Sulmona-L’Aquila, L’Aquila, Italy

**Keywords:** Musculoskeletal imaging, Rheumatology, Computed tomography: magnetic resonance imaging, Shear wave elastography

## Abstract

In recent years, technological improvements allowed imaging modalities to become increasingly essential in achieving early and precise diagnoses in the field of rheumatic and musculoskeletal diseases (RMDs). To date, imaging examinations are routinely used in all steps of diagnostic and therapeutic care pathways of patients affected by RMDs. The articles published in this Article Collection clearly show the efforts of researchers to find innovative applications of musculoskeletal imaging in clinical practice and to face the crucial challenges that remain in the interpretation and quality control of images. Highly performing diagnostic technologies are currently available to early diagnose and accurately monitor several musculoskeletal disorders, but also to guide personalized interventional therapeutic procedures tailored to the individual patients in the emerging process of precision medicine. Among these new modalities, some are particularly promising and thereby subject to several lines of research in RMDs, including SPECT-CT and dual-energy CT, MRI sequences, high and ultra-high frequency ultrasound with effective tools like shear wave elastography.

## Main text

Technological improvements have made imaging modalities increasingly essential to achieve early and precise diagnosis in the field of rheumatic and musculoskeletal diseases (RMDs). To date, imaging examinations are routinely used in all steps of diagnostic and therapeutic care pathways of patients affected by RMDs. The articles published in this Article Collection clearly show the efforts of researchers to find innovative applications of musculoskeletal imaging in clinical practice and to face the crucial challenges that remain in the interpretation and quality control of images. Indeed, highly performing diagnostic technologies are currently available to early diagnose and accurately monitor several musculoskeletal disorders [1–3], but also to guide personalized interventional therapeutic procedures tailored to the individual patients in the emerging process of precision medicine [4–7]. Among these new modalities, some are particularly promising and thereby subject to several lines of research in RMDs, including SPECT-CT and dual-energy CT [8, 9], novel MRI sequences [10–12], high and ultra-high frequency ultrasound with effective tools like shear wave elastography [13–16].

Notably, Takakura et al. compared the diagnostic performance of ultrasound and plain radiography in detecting fibula fractures in ankle sprain, showing similar accuracy with slightly increased sensitivity of ultrasound (94% vs. 81%) [17]. Still on the subject of ultrasound, Choate et al. tested the application of ultrasound with B-mode and power-Doppler to assess synovitis in surgically replaced joints of patients with rheumatoid arthritis reporting the reliability of this modality to detect and to monitor synovitis [18]. Shear wave elastography is increasingly investigated to evaluate the elastic properties of soft tissues in several settings. Indeed, according to the last guidelines of the European Society of Musculoskeletal Radiology, elastography should be considered as first choice level technique for Achilles tendinopathy, lateral and medial epicondylitis [19]. In confirmation of this, Otter and Colleagues compared thickness, vascularity, and shear wave elastography measures of the Achilles tendons of 24 patients with gout and 26 healthy subjects [13]. The authors found no differences in terms of thickness and vascularity, but significantly reduced stiffness in gout-patients. Furthermore, Gachon et al. plan a prospective study on pregnant patients to describe changes in the elastic properties of pelvic floor muscles through elastography providing potentially useful data concerning the risk of perineal tears at childbirth [14]. Ultrasound is also widely used and strongly recommended as a guidance for several interventional procedures in the musculoskeletal system [20]. In this Article Collection, a feasibility study on cadavers by Pape et al., proposed a novel approach in order to inject the coraco-humeral joint using ultrasound guidance to treat adhesive capsulitis, with interesting results that need to be subjected to prospective, clinical studies [5].

Some interesting papers on Single-photon emission computed tomography (SPECT)/CT have been published in this Collection. Dandois and Colleagues developed and validated a novel registration-based platform to compare SPECT/CT examinations performed before and after uni-condylar knee arthroplasty to accurately assess and locate changes in osteoblastic activity [21]. Tabotta et al. reported the improved diagnostic performance of SPECT/CT (87% sensitivity, 92% specificity) compared with that of bone scintigraphy in detecting prostate cancer bone metastases in 26 patients, thus highlighting the added value of the former to distinguish neoplastic locations from osteoarthritic changes [22]. Moreover, Wang et al. have nicely discussed about the attractive application of dual-energy CT to detect monosodium urate deposition in a rare pathologic condition of the spine, namely axial gout [9].

Concerning MRI, several papers are included in this Article Collection [10, 23–29]. Wuenneman et al. tested the performance of a novel 3D-MEDIC, i.e. multi-Echo-data-image-combination, sequence to identify superior labral anterior to posterior (SLAP) lesions [10]. Although gradient-echo sequences are generally less used in routine shoulder MRI protocols on high-field scanners [30], the 3D sequence proposed by the authors appeared to improve the accuracy of the non-arthrography MRI scan in detecting SLAP lesions, especially increasing the specificity when combined with 2D-fat-suppressed proton-density weighted images [10]. Harkey et al. used a semi-automated program to assess cumulative damage (tibio-femoral cartilage and bone marrow lesion) and disease activity (effusion-synovitis) of knee osteoarthritis using MRI [25]. These authors found that these composite metrics might be used as predictors of progression of knee osteoarthritis. Similarly, Aoki and Colleagues developed a fully automatic software to obtain a three-dimensional quantification of cartilage thickness/volume and meniscal extrusion reporting a relationship between medial tibial cartilage measurements and medial meniscus extrusion [27]. In keeping with artificial intelligence applications, the paper by Ishimoto et al. reported the potential application of a machine-learning automated system to accurately classify central lumbar spine stenosis using axial views of 4855 intervertebral levels taken from 971 lumbar spine MRI examinations [23].

Overall, the papers published in this Issue represent the emerging direction of research in musculoskeletal imaging toward a more personalized medicine approach aimed to a timely and more reliable diagnosis, accurate planning of tailored therapy, helpful prognostic imaging biomarkers, and thorough response assessment, moving from the traditional qualitative to quantitative images analysis. Physicians are called to keep up with the impressive advancements of diagnostic technologies to take full advantage and make best use of the huge potential of all imaging modalities.

The remarkable technological advancements driving the clinical utility of imaging RMDs resulted in zealous discussions by our Editors during peer-review, so we felt it necessary to further pursue this topic. We announce a Call for Papers on Digital health technologies in musculoskeletal care. Submissions on topics such as data science, wearable smart devices, and eHealth are welcomed.

## Data Availability

N/R.

## Abbreviations

3D-MEDIC: Multi-Echo-data-image-combination
CT: Computed tomography
MRI: Magnetic resonance imaging
RMDs: Rheumatic and musculoskeletal diseases
SLAP: Superior labral anterior to posterior
SPECT: Single-photon emission computed tomography
